# Open Online Courses for Strengthening Laboratory-Based Detection of Antimicrobial Resistance in Pakistan

**DOI:** 10.3389/fpubh.2022.773704

**Published:** 2022-03-15

**Authors:** Noureen Saeed, Mohammad Zeeshan, Joveria Farooqi, Sadia Shakoor, Kausar Jabeen, Faisal Riaz Malik, Jason Rao, Rumina Hasan

**Affiliations:** ^1^Department of Pathology and Laboratory Medicine, Aga Khan University, Karachi, Pakistan; ^2^Health Security Partners, Washington, DC, United States; ^3^London School of Hygiene and Tropical Medicine, London, United Kingdom

**Keywords:** open online course, antimicrobial resistance, antimicrobial sensitivity testing, laboratory strengthening, laboratory practice, resource limited settings

## Abstract

**Introduction:**

Quality-assured antimicrobial susceptibility testing (AST) depends upon the knowledge and skills of laboratory staff. In many low- and middle-income countries (LMICs), including Pakistan, such types of knowledge and skills are limited. Therefore, the objective of this study was to use openaccess online courses to improve the knowledge of laboratory staff involved in the detection and reporting of antimicrobial resistance (AMR).

**Methodology:**

Seven online modules comprising 22 courses aimed at strengthening the laboratory detection of Antimicrobial resistance (AMR) were developed. The courses were uploaded onto the website www.parn.org.pk. Participants had an option of selecting courses of their interest. Online registration and completion of a pre-course assessment (pre-test) were essential for enrolment. However, participation in post-course assessment (post-test) was optional. The number of registered participants and the proportion of participants who completed each course were computed. A paired *t*-test was used to assess the increase between mean pre- and post-test scores. The association between the participants working in public vs. private laboratories and course completion rates were determined using the chi-square test.

**Results:**

A total of 227 participants from Pakistan (March 2018 to June 2020) were registered. The largest number of registered participants and the highest completion rate were noted for AST and biosafety courses, while quality-related courses attracted a lower interest. A comparison of pre- and post-test performance using the paired mean score for the individual courses showed a statistically significant (the value of *p* < 0.05) improvement in 13/20 assessed courses. A higher course completion rate was observed in participants from public vs. private sector laboratories (56.8 vs. 30.8%, the value of *p* = 0.005).

**Conclusions:**

Our study suggests a promising potential for open online courses (OOCs) toward addressing knowledge gaps in laboratory practice in resource limited settings.

## Introduction

The attainment of sustainable development goals (SDGs) by all member states of the United Nations (UN) is a priority for a peaceful global future. However, there are multiple challenges and barriers in achieving these goals. The intention of the Health Goal (SDG3) is to ensure healthy lives and promote well-being for all, and, at all ages. Antimicrobial resistance (AMR) has emerged as an influencing factor that has affected society and healthcare and threatens the achievement of health-related goals (1).

The situation of AMR in Pakistan is not different from that in the rest of the world. Among Asian countries, Pakistan stands the highest in self-medication (2). Injudicious antibiotic usage and inadequate infection control are the major determinants for rapidly developing resistance (3). Antibiotics are prescribed on patients' demand. Additionally, over-the-counter purchase without prescription encourages self-medication practices; furthermore, aggressive antimicrobial use in agriculture, including farming, animal feeds, and fisheries, further compounds the situation. Another important contributing factor is the scarcity of trained and knowledgeable technical staff and limited laboratories with the capacity to detect and confirm quality-assured antibiotic resistance (4, 5).

Diagnostic technologies for clinical microbiology have seen rapid advances (6). These advances, however, also necessitate opportunities for updating the knowledge and skills of laboratory personnel (7). Clinical laboratories in resource limited settings face significant infrastructural challenges precluding them from benefiting from new developments and advances. Critical among such challenges is the limited availability of structured trainings for staff, a paucity of trained personnel and poor access to knowledge updates (8). These gaps contribute greatly to poor quality reporting, impact patient management, and add to the spread of AMR (9).

Internet-based learning and open online courses (OOCs) are increasingly being used for educational purposes (10–12), offering flexibility, portability, and in the case of OOCs without any financial constraints. Online microbiology learning has been shown to contribute to a better understanding of the subject and to improve knowledge about laboratory procedures (13–17). OOCs have also been used to strengthen antimicrobial stewardship (18). In recent times, OOCs have been widely used to share information on SARS-CoV2 and COVID-19 (19), and for laboratory staff, it is proposed as a valuable learning tool in settings where courses and structured on-job training program are scarce (20).

We have previously reported a significant improvement in the performance of microbiology laboratories in Pakistan following the training (workshops and practical sessions) of their staff. While that initiative was able to engage staff from 30 laboratories, including a cohort of 5 laboratories that were mentored over a 15-month period, the project was limited in its reach (21). A need to further expand the access to such training using easily available low-cost tools was identified. Our website www.parn.org.pk has been serving as a forum for sharing microbiology-related information and updates since 2007. We, therefore, aimed to develop OOCs for clinical microbiology technical staff working in low resource settings toward improving the quality and accuracy of microbiology with a focus on the detection of AMR using www.parn.org.pk as a platform. Most of the currently available online courses addressing AMR are aimed at staff who have undergone 16–18 years of education as well as structured laboratory training. In many LMICs, however, laboratory staff are enrolled after 10 or at most 14 years of education and acquire skills through on-job (often poorly supervised) work experience. As such, OOCs aimed specifically at this group of laboratory personnel are required. Our focus, therefore, was to use simple language supported by a strong emphasis on visuals and pictorials toward strengthening communication, and increasing reach. Participant learning was evaluated using a pre- and post-course assessment tool implemented as part of the OOCs.

## Methods

This initiative was conceived and planned to improve the skills of clinical microbiology technical staff working in laboratories with limited training resources. A primary and essential objective was to provide updated information on practices used in the detection of AMR. We further aimed to explore the use of OOCs as a strategy to increase access.

### Course Development

A dedicated full-time faculty was hired to develop course content, gather relevant and authentic information, improve website, and to disseminate course information on social media (e.g., Facebook and Twitter). They were further tasked with developing a liaison with faculty members and OOC participants for the prompt reply of queries, and, with web developers, to ensure active and smooth course development and participant involvement.

Multiple brainstorming sessions were held by the investigators who are teaching faculties and consultants of clinical microbiologists with experience in training medical students, residents, and technical staff. Based on our experience of working in the country and on the needs identified during our earlier study, courses were developed in areas deemed to be critical for strengthening the laboratory-based detection of AMR in the country and included; Antimicrobial Susceptibility Testing (AST), Biosafety in Clinical Laboratory, Interpreting CLSI Guidelines, Storage of Reference Strains, Laboratory Quality Management System, Antibiogram, Specimen Collection and Transport, and Non-culture-based techniques. These courses were divided into seven modules, with each module comprising a number of courses as described in Table 1.

**Table 1:** List of modules and the courses offered.

**Module objectives**	**Courses**	**Course description**
**1. Antimicrobial Susceptibility Testing (AST)**		
Objectives: • To explain the principles for performance, interpretation, and standardized reporting of AST	Good laboratory practices for AST	Describes practices to ensure safe, reliable, reproducible and quality laboratory results
• To define the concept of quality assured AST in accordance with international standards and best practices	Culture media used in AST	Discusses types of culture media used for antimicrobial susceptibility testing
• To share recent advances in performance of AST	Disc diffusion method for AST	Describes and demonstrates method for performing AST using disc diffusion method
	MIC by agar dilution and E test methods	Describes and demonstrates performance of minimum inhibitory concentration using agar and Epsilometer (E-test) based methods
	MIC by broth dilution method	Describes and demonstrates performance of minimum inhibitory concentration using broth dilution method
	Recent advances in AST	Discusses recent innovations and automation in antimicrobial susceptibility testing
**2. Biosafety in the clinical laboratory**		
Objectives: • To define important terminologies used as part of Good Laboratory Practices (GLP)	Good Laboratory Practice and waste management	Principals of good laboratory practices including safe management and disposal of laboratory waste shared
• Define waste management and waste segregation	Biosafety cabinets	Explains uses of different classes of biosafety cabinets
• Describe the working of different classes of biosafety cabinets • List the bio-risk groups of microorganisms and their handling in the laboratory	Risk Groups of Microorganisms and biosafety levels	Lists categories of microorganisms based on pathogenicity and biosafety level facilities required for their handling
**3. Interpreting CLSI guidelines, ATCC storage, revival and subculture**		
Objectives: • To emphasize importance of standardization of AST	AST quality standards	Explains international guidelines for selecting AST methods, result interpretation and reporting
• Provide guidance for interpretation of susceptible/ resistant categories	Quality control strains	Discusses uses and role of control organisms recommended in performing AST procedures
• Define procedure for storing and sub-culturing quality control strains	ATCC strains storage and revival guidelines	Outlines standardized storage and procedure for sub-culturing of quality control strains toward preventing loss of strain viability
**4. Laboratory quality management system**		
Objectives: • Describe the WHO model of laboratory quality management system	Introduction to quality management system	Describes definition of quality management system and categories of processes involved
• Outline components of quality management system	Quality management system part II	Details organization, quality control procedures and quality indicators
	Quality management system part III	Explains record keeping, documentation, errors and incident management
**5. Antibiogram**		
Objectives: • Emphasize the importance of antibiogram • Provide guidance for their preparation	Antibiogram and their preparation	Discusses the principles and uses of antibiogram and describes steps involved in their preparation
**6. Specimen collection, transport, and processing**		
Objectives: • Explain the essentials of specimen collection and transport	Collection of specimens for bacterial culture	Describes the appropriate procedures required in collection of samples for bacterial culture
• Guidance for processing of clinical specimens	Transport of specimen and receipt in the laboratory	Emphasizes the need for safe and rapid transport of clinical specimens from point of collection to clinical laboratories. Specimen acceptance and rejection guidelines are discussed.
• Describe identification of pathogens	Processing of specimens for bacterial culture	Describes the steps used in processing specimen for bacterial culture and outlines the procedures for different specimen types.
	Identification of pathogens and reporting culture results	Describes methods used in identification of pathogens. Basic principles for culture and sensitivity reporting are outlined.
**7. Non-culture-based techniques**		
Objectives: • Define the principles of non-culture-based techniques used in bacteriology	Nonculture based techniques part I	Defines serological techniques for diagnosis of bacterial infections
• Describe diagnostic approaches for different infections	Nonculture-based technique part II	

To ensure standardization and authenticity, teaching material was adapted from recent guidelines and manuals, including the Clinical and Laboratory Standard Institute (22) and the American Society of Microbiology procedure manual (23). The developed teaching material and the details of used contents were extensively discussed by the investigators before it was incorporated into a video script for uploading onto the website. An internal review process was implemented in which university faculties with a special interest in educational development and in online education were involved to evaluate and ensure content quality. Input and suggestions about course contents were also taken from senior laboratory technical staff.

The entire content was in English; however, all efforts were made to keep the language simple and to provide visual support so that the content could be easily understood by participants with limited command of the English language. To maximize learning, textual information was consolidated with in-house pictures, animations, and videos. All relevant pictures, including video with script and graphics, were developed and prepared in-house by professional photographers and an audiovisual (AV) team. To ensure the quality of video materials and to avoid editing errors, post-production reviewing and voice-over were carried out by a dedicated faculty in collaboration with the AV team.

### Administration of the Courses

The website www.parn.org.pk, which was established by our group earlier, was used as a platform for OOC dissemination. Pakistan Antimicrobial Resistance Network (PARN) was conceived and developed by our group members in 2006 with the aim of sharing updates and information on AMR. The PARN website was upgraded for better performance and data retrieval with the help of professional website developers. An online OOC section was added in the www.parn.org menu, and relevant modules and related course contents were uploaded.

Before it was accessible on the website, the project was piloted, and the courses were attempted by the technical staff of 10 laboratories to ensure content clarity and understanding.

Online modules were shared between March 2018 and December 2018 with new courses uploaded onto the website every fortnight. These were open courses, and all interested participants could register and access course contents and quizzes. Participant registration required a one-time enrolment to provide (email and password linked) access to all the modules and related courses on offer. At the time of registration, participants have requested information about their location (city and town) and their association with laboratories (whether public or private).

The option to select and complete the courses of their interest was available to all participants. All courses were independent and did not require the completion of a preceding course. There was no specific time duration for course completion, and participants could complete at their own pace.

### Learning Assessment

To assess learning, a pre-course assessment (pre-test) and a post-course assessment (post-test) were developed for every offered course. While pre- and post-tests were non-identical, each consisted of 5–10 questions (included true/false, and best choice questions). The questions included were content-based and prepared and screened by the faculties who were part of the study team. Ambiguous questions were deleted. Prior to being uploaded on the website, the questions were further reviewed by a faculty with expertise in education.

Following the registration, the access to course content was conditional to online completion of the pre-test. Participation in the post-test through optional was required for the award of completion certificate. All questions included in the assessments were scored individually. All questions were awarded equal marks. Scores were generated automatically. An increase in the post-test score as compared to the pre-test score was used as a reflection of knowledge acquisition by the participant.

### Data Saving and Retrieval

The database of course registration, tests attempted, and scores of individual participants were saved on a Structured Query Language (SQL) server of the website provider. Back-up of this data was saved on an external dedicated hard drive monthly. Excel spreadsheet was used to store the retrieved data. Data on participants' city/town of origin were grouped in line with the provinces of Pakistan and classified as urban or rural according to the population size of their towns/cities (24). The participants' affiliation with public or private sector laboratories was also recorded.

Universal sampling was applied, and data of participants enrolled between March 2019 and June 2020 were retrieved for analysis.

### Statistical Analysis

The retrieved raw data from the website were saved in excel sheets. It was then cleaned, coded, and transferred to SPSS, IBM version 21.0 for analysis.

Frequencies of participants' characteristics, courses attended and completed were calculated. Mean pre-test scores were compared to mean post-test scores for each course using a paired *t*-test. The association between public and private sectors and participant origin, course attendance, and completion rates was determined using the Chi-square and Fisher exact tests, as appropriate.

### Patient and Public Involvement

Neither patients were involved in setting the research question or the outcome measures nor were they involved in developing plans for the recruitment, design, or implementation of the study. However, research was shared in national and international conferences and social media for public involvement.

## Results

### Participation and Course Preference

A total of 227 individual participants were registered for the 22 courses offered as part of these OOCs with 66 (29%) registering for more than one course. Of the total 22 courses offered, 9 attracted more than 25 participants each (Table 2) with the highest participation 72.5% (*n* = 165) in the course on AST. The most popular modules in terms of registered participants were the ones related to AST and to Biosafety in Clinical Laboratories. On the other hand, quality-related courses; those linked to the quality of AST and on laboratory quality management attracted a much lower interest (Table 2). The overall completion rate, as assessed by the number of participants opting for the post-course test, ranged between 31.5 and 90%. A high completion rate (of more than 70%) was noted in 3/6 courses of the AST modules, and 2/3 courses were included in the Biosafety in Clinical Laboratory modules.

**Table 2:** Enrolment frequency and completion rates for different courses and an improvement in pre- and post-course test scores among different courses are shown below.

**No**.	**Courses**	**Number of participants registered**	Number of participants who completed course (%) **	**Pre- test mean (SD) score**	**Post-test mean (SD) score**	**Paired mean differences**	**95% CI of the difference**	***P*-value**
**Module 1. Antimicrobial Susceptibility Testing (AST)**								
1	Good laboratory practices for AST	165	77 (46.7)	56.7 (28.6)	83.7 (17.1)	26.93	20.73–33.14	<0.001*
2	Culture media used in AST	35	31 (88.6)	46.0 (35.7)	85.7 (20.1)	39.65	26.29–53.00	<0.001*
3	Disc diffusion method for AST	50	30 (60.0)	58.3 (28.0))	81.3 (29.6)	23.07	11.65–34.49	<0.001*
4	MIC by agar dilution and E test methods	36	25 (69.4)	48.8 (31.7)	89.6 (20.9)	40.80	27.01–54.59	<0.001*
5	MIC by broth dilution method	29	24 (82.8)	54.2 (38.9)	92.5 (17.5)	38.33	22.80–53.87	<0.001*
6	Recent advances in AST	31	25 (80.6)	35.2 (40.9)	87.2 (22.3)	52.00	33.24–70.77	<0.001*
**Module 2. Biosafety in clinical laboratory**								
1	GLP and waste management	54	17 (31.5)	41.8 (16.7)	86.2 (17.9)	44.47	30.49–58.46	<0.001*
2	Biosafety Cabinets	46	33 (71.7)	46.7 (35.9)	87.9 (22.9)	41.21	28.81–53.62	<0.001*
3	Microorganisms risk groups & biosafety levels	30	27 (90.0)	58.1 (30.6)	87.7 (18.3)	29.63	18.20–41.06	<0.001*
**Module 3. Interpret CLSI guidelines, ATCC storage, revival and subculture**								
1	AST quality standards	7	6 (85.7)	26.7 (37.2)	81.7 (24.0)	55.00	12.11–97.89	0.022*
2	Quality control strains	6	4 (66.7)	40.0 (40.0)	95.0 (10.0)	55.00	−5.24–115.24	0.062
3	ATCC strains storage, revival, and subculture guidelines	8	5 (62.5)	36.0 (35.8)	84.0 (20.7)	48.00	7.38–88.62	0.030*
**Module 4. Laboratory quality management system**								
1	Introduction to quality management system	9	5 (55.6)	64.0 (26.8)	78.0 (22.8)	14.00	−30.42–58.42	0.431
2	Quality management system II	6	4 (66.7)	65.0 (30.0)	83.0 (22.7)	18.00	−54.85–90.85	0.489
3	Quality management system III	6	5 (83.3)	48.0 (22.8)	86.0 (16.7)	38.00	19.58–56.42	0.005*
**Module 5. Antibiogram**								
1	Antibiogram	7	4 (57.1)	25.0 (43.6)	97.5 (5.0)	72.50	5.78–139.22	0.041*
**Module 6. Specimen collection, transport and processing**								
1	Collection of specimens for bacterial culture	7	5 (71.4)	60.0 (29.2)	84.0 (26.1)	24.00	−17.74–65.74	0.186
2	Transport of specimens for bacterial culture	2	2 (100)	65.0 (21.2)	90.0 (14.1)	25.00	−292.66–342.66	0.500
3	Specimen processing for bacterial culture	2	1 (50.0)	80 (28.3)	100 (-)	20.00	-	-
4	Identification of pathogens and reporting culture results	3	2 (66.7)	55.0 (21.2)	100 (0)	45.00	−145.59–235.59	0.205
**Module 7. Non-culture-based techniques**								
1	Non-culture-based techniques I	2	2 (100)	60 (0)	80 (0)	20.00	-	-
2	Non-culture- based techniques II	2	2 (100)	70.0 (28.3)	80.0 (0)	10.00	−244.12–264.12	0.705

*Courses were completed by less than two participants, and no variance in scores was excluded from a statistical analysis*.

*The courses achieved a statistically significant improvement in mean test scores.

**Participation in the post-course test was used as a measure of course completion.

### Improvement in Scores

The mean pre- and post-course test score comparison showed a statistically significant improvement in 13/20 courses that could be assessed. These included all 6 courses of the AST module and all 3 courses of the Biosafety in Clinical Laboratory module (Table 2). The courses wherein the difference did not reach significance were mostly those in which the number of participants completing the post-course test were 5 or less (Table 2).

### Outreach

**Figure 1:**
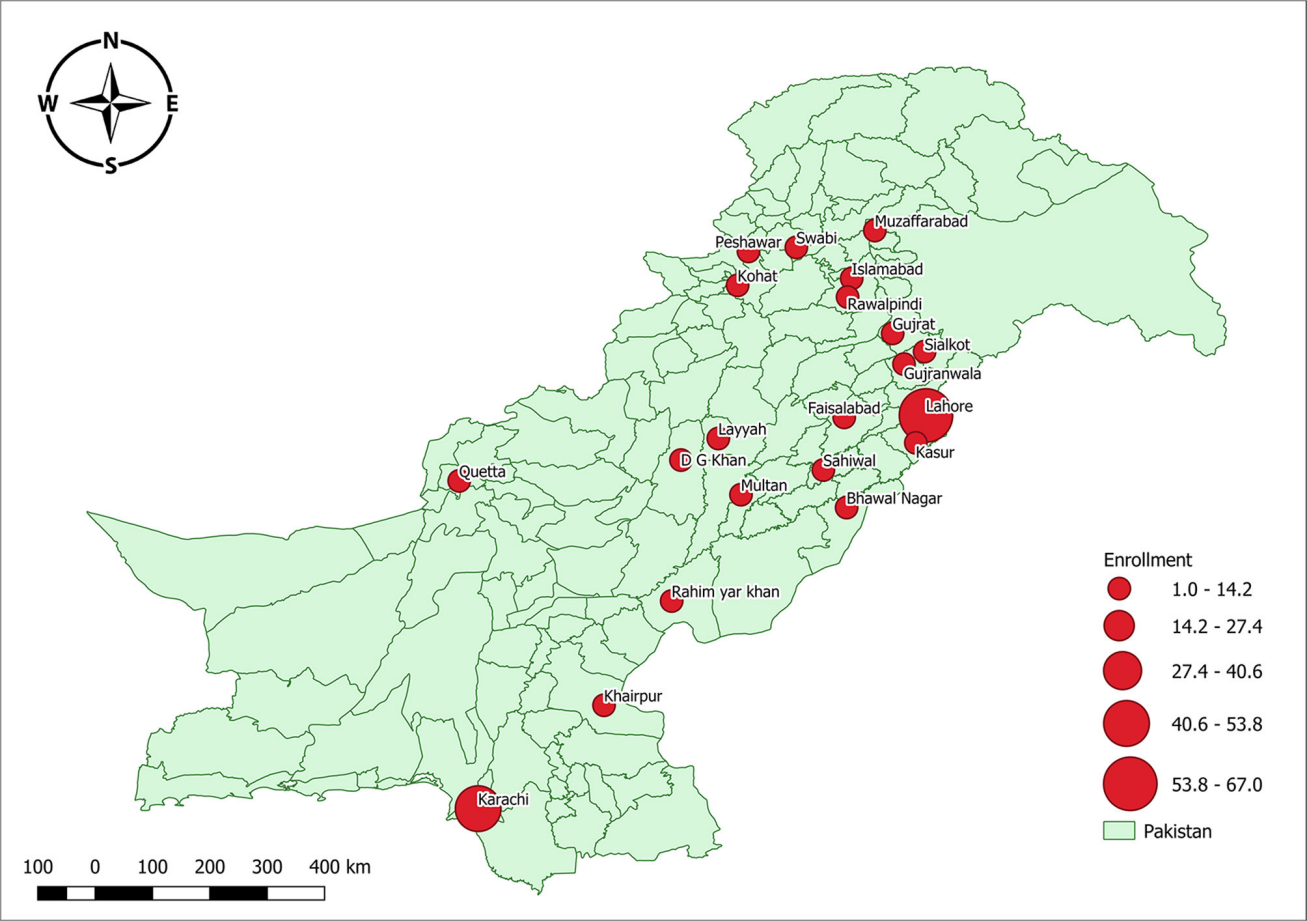
Microbiology massive online open courses (MOOCs) participation from the different parts of the country.

The reach of OOCs was assessed using participant information on location and affiliation. The data on geographic location were only available for 163 participants and 157 for affiliation. Of the 163 participants that provided location information, 92.6% of the participants belonged to the urban areas, 56.4% belonged to Punjab, 32.5% to Sindh, 5.5% KPK, 3.1% to Federal Capital, and only 0.6% were from Baluchistan. Figure 1 shows the cities with a marker size proportionate to the number of participants belonging to them.

### Differences Across the Public and Private Sector

Data from the 157 participants whose affiliation was available showed that 75.2% belonged to the public sector while 24.8% were working in the private sector. The assessment of OOC penetration across the private and public sector indicated a statistically significant difference between Punjab and Sindh, with more public sector attendees in Punjab as opposed to in Sindh (Table 3). Our data further indicated greater participation and completion from public sector participants vs. those affiliated with the private sector (Table 3).

**Table 3:** Open online course (OOC) penetration and completion across public and private sectors: details of 157 participants working in laboratories in Pakistan.

**Participant distribution**	**Public n (%)**	**Private n (%)**	**Chi-square *p*-value**
Demographics*	***N*** **=** **115**	***N*** **=** **37**	
Urban	104 (90.4)	37 (100)	0.051
Rural	11 (9.6)	0 (0)	
**Provinces**	***N*** **=** **115**	***N*** **=** **37**	
Punjab	82 (71.3)	7 (18.9)	<0.001^¥^
Sindh	23 (20)	26 (70.3)	
Baluchistan	1 (0.9)	0 (0)	
KPK	5 (4.3)	3 (11.1)	
AJK	1 (0.9)	0 (0)	
Federal Capital	2 (1.7)	1 (2.7)	
Abroad	1 (0.9)	0 (0)	
**Courses attended**	***N*** **=** **118**	***N*** **=** **39**	
**Overall completion rate**	**67 (56.8)**	**12 (30.8)**	**0.005** ^¥^
**Antimicrobial Susceptibility Testing (AST)**			
Good laboratory practices in AST	80 (67.8)	29 (74.4)	0.441
Completion	57 (71.2)	5 (17.2)	<0.001^¥^
Culture media used in AST	29 (24.6)	5 (12.8)	0.122
Completion	28 (96.6)	2 (40.0)	<0.001^¥^
AST by disc diffusion method	31 (26.3)	10 (25.6)	0.938
Completion	23 (74.2)	3 (30.0)	0.012^¥^
MIC by Agar dilution and E-test method	29 (24.6)	4 (10.3)	0.057
Completion**	24 (82.8)	0 (0)	0.003^¥^
MIC by broth dilution method	25 (21.2)	3 (7.7)	0.056
Completion**	23 (92.0)	0 (0)	0.003^¥^
Recent advances in AST	27 (22.9)	3 (7.7)	0.036^¥^
Completion	23 (85.2)	1 (33.3)	0.033^¥^
**Biosafety in clinical laboratory**			
Good laboratory practices and waste management	45 (38.1)	4 (10.3)	0.001^¥^
Completion**	16 (35.6)	0 (0)	0.289
Biosafety cabinets	41 (34.7)	3 (7.7)	0.001^¥^
Completion	31 (75.6)	1 (33.3)	0.112
Organisms risk groups and biosafety levels	27 (22.9)	2 (5.1)	0.013^¥^
Completion**	25 (92.6)	2 (100)	1.000
**Quality management systems (I, II, III)**	6 (5.1)	1 (2.6)	0.508
Completion**	5 (83.3)	1 (100)	1.000

*Data from the 157 participants whose affiliation was available were included.

**Fisher exact test was run instead of chi-square due to empty cells in a 2 × 2 table.

*^¥^Achieved a statistical significance*.

*The text in bold represents the total counts and overall rates for the respective categories and the rows below them represent the subcategory distributions*.

## Discussion

While students in Pakistan are familiar with OOCs with more than 90,000 students registered for online courses provided by several high-ranking global universities, the majority of these are students who are seeking degrees (25). The extent of the use of OOCs in the country as a tool to update knowledge is not known. As such, our study reported the registration of 227 participants in the different courses aimed at improving laboratory technical expertise is very encouraging.

It was interesting to note that the preferred courses were the ones focusing on antimicrobial sensitivity testing (AST) and on Biosafety. The interest in antimicrobial sensitivity testing updates may have been sparked by the emphasis on quality-assured AST being discussed in earlier workshops (21), through sessions organized by the Medical Microbiology and Infectious Diseases Society of Pakistan (https://www.mmidsp.com/) and by the declaration of AMR as a national priority (26). Similarly, biosafety awareness is likely to have been created through the numerous activities led by the Pakistan Biosafety Association (http://pbsa.org.pk/) as well as global interest in biosafety.

Pakistan national accreditation council (PNAC) certifies clinical laboratories in accordance with ISO 15189 standards; however, accreditation is not mandatory and the participation of laboratories in accreditation remains voluntary (27). While the licensing of laboratories and laboratory workers is recommended, implementation modalities are yet to be being developed (28). As a result, quality standards practiced in microbiology laboratories across the country are not uniform. The limited interest of participants in courses focusing on quality systems in this study suggests that quality is likely not prioritized and highlights a need for more efforts toward advocating for quality standards among laboratory personnel. We hypothesized that greater advocacy for self-improvement and continuing education, combined with prioritizing key topics, will encourage a greater interest in these areas.

A global antimicrobial stewardship course run as a massive online open course (MOOC) reached a massive 32,944 people from 163 countries. Of these, 33–37% completed at least one of the steps in the course (18). Other studies report a 96% dropout rate in OOCs over a period of 5 years (29). Failure to complete modules/courses is attributed to a number of reasons, including the level of motivation, work experience, the lack of time, insufficient background knowledge, inability to understand the course, or unavailability of support/help (30). Using attempts at the post-assessment test as a measure of completion, a completion rate of between 31.5 and 90% among participants registered for the different courses in this study is encouraging and suggests that our target audience is self-motivated. The comparison of test scores for individual participants showed that in 13/20 courses that could be assessed, the completion of the course resulted in a significant improvement in performance as compared to pre-course scores. These findings suggest that OOCs were considered as an effective teaching strategy, at least in the short term, for most of the offered courses. Although we acknowledge that in the long run, course evaluation and a survey pertaining to how much of the knowledge gained was introduced into practice would be a better measure of its impact on practice.

With 92.6% of the participants being based in urban centers, our data show a strong urban bias. To some extent, this may be attributed to fewer laboratories in rural areas. It is however also likely to be a reflection of the fact that online education is still a relatively uncommon mode of learning in the rural and far-flung areas. To an extent, the difference may be attributed to limited awareness; however, it may also be attributed to technical challenges and limited resources in rural centers, including a low literacy rate, a poor infrastructure, language barriers, limited access to personal computers, power outages, and limited internet users.

These OOCs attracted participation from across the country. In accordance with the population distribution of the country, the highest number of participants were from the provinces of Punjab followed by Sindh. In Punjab, the highest participation and completion rate (71.3%) were from among public sector participants, while in Sindh 70.3% participants were from the private sector. This difference is difficult to explain and needs further exploration. Overall, of the 157 registered participants whose affiliation was available, 115 were from the public sector laboratories. While it is encouraging to see a strong interest from public sector participants, relatively low participation from individuals working in the private sector is concerning. Private sector laboratories are reported to handle at least 50% of testing in Pakistan. A poor representation of learners from this important segment of health service providers is concerning. While it is possible that a few private laboratories may provide internal training for their staff, by enlarging the capacity of the majority of private laboratories in the country to offer such training that is limited. As such, low participation of staff from this sector suggests either poor access to online training opportunities or that such training are of a lower priority for private laboratory. The latter has significant implications in terms of the reliability of laboratory results for our population. As such, exploring modalities for increasing engagement and engagement of the private sector would be important.

Overall, the interest as well as an improvement in the knowledge of laboratory personnel who participated in the courses are encouraging and call for an expansion of such teaching and training modalities. Our study indicates that OOCs supported by visuals, graphics, and animations are considered as a useful tool for their use across countries where English is not the first language. Those such learning opportunities are much needed and could contribute greatly toward strengthening clinical microbiology laboratories not only in Pakistan, but also across other resource limited settings.

Moreover, interventions, such as those that improve diagnostic microbiology practices, would further contribute toward strengthening hospital-based stewardship programs, e.g., through the implementation of appropriate diagnostic tools and the interpretation of results (31, 32), selective reporting of AST results, customizing susceptibility reports to be consistent with local infectious disease treatment guidelines, and, including comments in microbiology reports that facilitate clinicians to delineate between pathogens, colonization, or contamination (33, 34).

To conclude, the laboratory detection of AMR is a multifaced approach, including susceptibility testing, quality control, and the interpretation of the result as the essential steps. Our analysis suggests that while OOC participants showed considerable interest in the AST module, their interest in other relevant modules relating to quality control, the interpretation of the CLSI guideline, laboratory quality management, and specimen collection was, however, limited. Comprehensive efforts toward increasing the focus on the quality aspects of laboratory testing are required to ensure reliable results in LMICs.

Public and private laboratories are important service providers in Pakistan (35, 36). However, as in many LMICs, national training efforts focus primarily on the public sector. To strengthen capacity nationwide, it is vital to also regularly upscale and monitor the technical skills of private providers, e.g., through national platforms. Compulsory accreditation of diagnostic laboratories through in-country/international accrediting bodies would further strengthen compliance with quality standards.

## Limitations of this Study

Due to hitches in the program, some data related to initial participants could not be recovered, as such only data that could be recovered and analyzed are presented. Additionally, while online courses require uninterrupted power supply and internet facilities, continuous power supply and good quality internet are the major concerns in Pakistan. There is, therefore, a possibility that some participants facing connectivity issues may have lost interest and did not attempt to complete the sessions.

The status of computer literacy in participants was not evaluated, and therefore it is not possible to assess whether they have taken any assistance while attempting test questions.

Finally, while www.parn.org.pk does attract global visitors, the number of international participants for OOCs courses was limited with a total of 5 participants registered from 3 countries (Nigeria, Algeria, and Saudi Arabia). Therefore, due to low numbers, international participants were not included in the presented data.

## Data Availability Statement

The raw data supporting the conclusions of this article will be made available by the authors, without undue reservation.

## Ethics Statement

This study was approved by the Ethical Review Committee Aga Khan University, Karachi, Pakistan (ERC# 3487-Pat-ERC-15).

## Author Contributions

SS, JR, and RH conceived the study. NS, MZ, JF, KJ, SS, and RH contributed to the development of course material. NS, MZ, and FM involved in preparing the audio–video material and uploading it online. NS, MZ, JF, FM, and RH helped in data retrieval, compilation, and analysis. NS, MZ, JF, SS, KJ, FM, JR, and RH contributed to the preparation and approval of this manuscript. RH is the guarantor. All authors contributed to the article and approved the submitted version.

## Funding

This study was supported by the Health Security Partners (HSP)-USA, as part of their collaboration with the Department of Pathology and Laboratory Medicine, Aga Khan University, for laboratory capacity building, and strengthening for AMR surveillance.

## Conflict of Interest

The authors declare that the research was conducted in the absence of any commercial or financial relationships that could be construed as a potential conflict of interest.

## Publisher's Note

All claims expressed in this article are solely those of the authors and do not necessarily represent those of their affiliated organizations, or those of the publisher, the editors and the reviewers. Any product that may be evaluated in this article, or claim that may be made by its manufacturer, is not guaranteed or endorsed by the publisher.
